# Managing complexity in research data management for heterogeneous biotechnological data: a perspective on interacting as data steward

**DOI:** 10.1007/s00449-026-03380-0

**Published:** 2026-07-20

**Authors:** Martina Rehnert, Ralf Takors

**Affiliations:** Institute of Biochemical Engineering, University of Stuttgart, 70569 Stuttgart, Germany

**Keywords:** Bioengineering, Bottom-up community approach, FAIR data responsibility

## Abstract

Biotechnology is an interconnected and globally significant field with expanding impact on industries, bioeconomy and healthcare. The associated rapid increase in data generation requires effective research data management (RDM) that ensures compliance with the FAIR principles (findable, accessible, interoperable and reusable) while addressing challenges related to heterogeneous data formats, metadata complexity, data protection, and governance requirements. Furthermore, archiving and storage of heterogeneous, often proprietary data formats, and subject-specific ontologies in cross-domains can pose challenges for RDM. In a use case involving 50 international, cross-discipline biotechnology researchers, an institutional repository was used for RDM and to manage heterogeneous data formats such as experimental, computer-aided modeling and process engineering data in accordance with the FAIR principles. The RDM built on the repository’s existing centralized infrastructure, but this did not entirely meet user expectations for their practical research applications. While centralized top-down infrastructures of repositories provided the technical foundation for FAIR data handling, our observation showed that generic repository frameworks and standardized workflows required continuous, discipline-specific, and human mediated support integrated into everyday research workflows. As intermediaries between institutional requirements and research practice, data stewards can provide valuable insights into the challenges of implementing sustainable RDM helping to optimize repository support structures and align them with researchers’ evolving needs in complex real-world biotechnology research environments.

## Challenges on bioengineering data management

The global bioengineering market is growing dynamically and is expected to be worth over 820 billion US dollars by 2029, reaching approximately 1,484 billion US dollars by 2035 [1]. Key drivers include innovations in synthetic biology, artificial intelligence (AI), precision medicine, recombinant DNA technology [1] and industrial scale-up [2]. The global biotech market, which includes biologics, gene therapies and diagnostics, is expected to grow robustly at a Compound Annual Growth Rate (CAGR) of 9.9% while the CAGR for artificial intelligence in pharma and biotech is expected to be 16.2% between 2025 and 2033 [3].

The associated exponential growth of biotechnology research data, driven by omics data, high-throughput sequencing, and process modeling, requires responsible, effective research data management (RDM) [4] with an infrastructure and potential for machine learning [5] and AI [6]. In this context, core processes, guidelines, human resources, and classifications are required [7]. Classifications are particularly needed in biotech to comprehensively link the two disciplines of science and technology with each other and their heterogeneous data [5]. Although this heterogeneous data is increasingly being combined for strain design, process optimization and scaling, its reusability and reproducibility remain limited due to insufficient standardization, incomplete metadata and absence of overarching uniformity [8].

Ontologies as formal structure provide standardized terms for describing datasets and ensure metadata uses controlled datasets to enable consistent, machine-readable annotations and standardized organization of experimental information to improve the interoperability and reusability [9]. Overlapping terms in ontologies or insufficiently detailed concepts [10] can result in ambiguous mappings, inconsistent annotations, and reduced interoperability and reproducibility [11]. Consequently, standardized registries and repositories [12], and harmonized metadata frameworks are essential to support consistent data integration and exchange. Achieving sustainable RDM further requires clear governance structures to guide researchers toward appropriate and community-recognized standards [13]. Together, technology, ontologies, frameworks, repositories and stewardship can promote the FAIR data principles (findability, accessibility, interoperability, reusability) [14] considered the gold standard for sustainable RDM [15].

However, technical infrastructure alone is insufficient, and need to be implemented by cultural and institutional environments [16]. Specific characteristics of biotechnology research must be taken into account when implementing the FAIR principles as core element. RDM has to be supported by adequate training, incentives and user-centered tools, that integrate into scientific workflow [17]. Publishing research data enhances transparency but can also give rise to unexpected vulnerabilities or dual-use application. The benefits of greater openness and data disclosure must therefore be weighed against potential risks. To address these competing priorities, it is necessary to examine how FAIR data principles are translated into responsible research practice across the data life cycle (DLC) and across disciplines, a topic that remains underexplored, particularly in biotechnology. Although embedded in international and EU frameworks (e.g., EU regulation 2021/821) [18], responsibilities are often underspecified, leaving researchers to interpret them in practice.

RDM, infrastructure, metadata, ontologies, registries, standardization and user commitment is not only a technical exercise [4] but a critical step towards building resilient, community driven FAIR data management [19]. In addition, this article presents a longitudinal, practice-based perspective, based on six years of experience providing operational RDM support within the DFG Priority Program SPP 2170 “InterZell” and an institutional use case with 50 researchers. We reflect on implementation experiences, operational challenges, and lessons learned while applying FAIR-oriented workflows in heterogeneous biotechnology research environments. Challenges such as the emergence of new data formats and workflows, evolving metadata requirements, and semantic shifts in ontologies are characteristics of rapidly changing scientific data ecosystems, and highlight the need for adaptable RDM strategies within biotechnology research. Using a biotechnology use case, we demonstrate, how governance and technical repository strategies can be combined with user-centered, demand-driven approach to support effective RDM.

### Strategic initiatives supporting the FAIRness

International initiatives such as the Research Data Alliance (RDA) [20] the European Open Science Cloud (EOSC) [21], and German national initiatives such as the NFDI [22] are increasingly advocating FAIR and trustworthy data exchange. Biotech data relating to experiments in life science research (genomics, microbial cells, metabolic data) and technology data (process data) are often very heterogeneous and are stored in project-specific, temporary, or fragmented databases [5]. NFDI initiatives therefore demand a standardized, networked, and sustainable digital infrastructure to provide FAIR research data across all disciplines [22]. NFDI provide standardized metadata or automated and systemic quality checks on data integrity through over 26 consortia like DataPlant [23] NFDI4Microbiota [24] NFD4Ing [25] and bridges the gap between academic research, researchers and industry-applicable data standards for long-term reuse and reproducibility [26].

### Findability and interoperability in cross-discipline research

In biotech, with cross-discipline research, heterogeneous data are complex to integrate. Applying metatags and defined ontologies for the formal description of biotech knowledge, concepts, terms, and their relationships helps organize, interpret and curate the increasing amount of data [9]. Ontology-based frameworks also play a role in biotechnology and synthetic biology in making biological models standardized, interoperable, and machine-readable by enabling consistent data annotation and exchange between different disciplines [26] like:

In synthetic biology, ontology linked standards such as SBOL (Synthetic Biology Open Language) [27] enable predictive modeling of engineered systems by connecting genetics and regulatory, metabolic and bioprocess context from metabolic flux changes, transcriptional regulation, metabolic effects, and feedback loops, improving predictive accuracy. This structured knowledge supports multiscale simulations and digital twin framework, where changes in gene expression or nutrient conditions of microbial cells dynamically propagate through cellular and bioreactor scale-up models [26].

 Simulations of complex systems such as metabolic pathways, gene regulation networks or bioreactor scale-up simulations require more than just technological availability [28]. Simulations without metatags and ontologies remain models that refer to a one-off situation and are not interoperable [29]. Simulations using computational fluid dynamics (CFD) generate extremely large data sets due to high-resolution meshes, multiple field variables, and transient time steps [30]. A major challenge in CFD is traceability, as simulated results depend on interconnected factors, that are often insufficient documented in metadata or belong to different ontologies which can compromise long term archiving and reproducibility. Furthermore, storing CFD simulations data is also an energetic question as related infrastructure and energy-dependent storage needs to be maintained ‘somewhere’ for decades. Ontology such as Systems Biology Ontology (SBO) [31], Gene Ontology [GO], or Chemical entities of Biochemical Interest (ChEBI) [32] provide standardized terms for genes, reactions or metabolites in biotech. By annotating simulation components with unique ontology identifiers, different models can recognize equivalent biological entities, enabling model reuse, integration, and automated composition across platforms. Ontologies and metatags enable simulations to become interoperable and go beyond isolated models [26].

### Accessibility and reuse in biotechnology

Publishers, such as Nature [13] and funding organizations (US NIH) [33] are requesting publications in connection to FAIR data management. However, data exchange is also subject to ethical considerations [34, 35] and legal regulations such as EU 2021/821 [18], also apply to data sharing, which makes it difficult to define general requirements and may lead to reservations about adopting FAIR due to legal uncertainties [36, 37]. Biotechnology research may entail dual-use risks, where findings could be misapplied for sensitive purposes, necessitating export controls and careful data sharing practices. Additional constraints arise from the need to protect sensitive personal data in compliance with data protection regulations, as well as from national strategic or regulatory restrictions. A pragmatic approach could be to implement controlled, case-by-case access with human oversight [34]. However, while the FAIR principles focus more on the technical aspects of data management [14], supplementing principles are introduced to address concerns about insufficient access controls and the practical implementation of FAIR in ethical [35], methodological, and organizational contexts [34, 38]. While CARE (Collective benefit, Authority to control, Responsibility, Ethics) [35] focus on ethical governance, TRUST (Transparency, Responsibility, User focus, Sustainability, Technology) [38] supports the sustainability of repositories and infrastructure, and socio-Technical dAta Protection frameworks (STAP) [39] are discussed. The CLEAR model advocates cognitive interoperability in order to influence human-actionable data and metadata [40].

### Responsibility and governance for research data

General [41], bio-specific repositories [42] or organizational repositories provide frameworks that support compliance with ethical and legal regulations in a secure top-down structure. However, they require user acceptance to create appropriate metadata and ensure compliance with regulations and access controls, which can be time- and resource-intensive and need support, consulting, storage capacity and financial support for infrastructure and human resources [12]. Researchers often limit data disclosure due to concerns about misinterpretation, reputation loss [36], unauthorized reuse [37], or competition [16] or often lack formal comprehensive training in law, AI, or data management [16].

There is frequently an absence of pragmatic models to assist researchers in handling sensitive information at an initial stage, implementing suitable access restrictions, clearly recording limitations, and choosing appropriate archives and licensing structures for efficient data administration at the start of the DLC to guarantee compliance with regulatory and FAIR principles [36, 37]. While most universities have the guidelines in place [43], it is still the responsibility of researchers to determine how their data is handled [16]. Due to dynamic changes and requirements, RDM must constantly adapt. Focusing RDM on the life cycle of data can help to ensure consistent compliance with regulatory and FAIR requirements when data is published and described in a trustworthy manner as part of a use case [43].

### Heterogeneous and responsible data in biotechnology research

In experimental biotechnology, data management requirements are often heterogeneous due to the field’s interdisciplinary nature, which brings together distinct research practice, data types, and standards from the life sciences and engineering [44]. In biomedicine and the life sciences, “Collective benefit, Authority to control, Responsibility, Ethics” (CARE) is increasingly being proposed as a framework for protecting privacy and security, including indigenous data [35].

While general repositories like Zenodo [41] are open to all data, there are also specific biotechnology repositories or databases for enzymes and proteins as data knowledge bases [45] or organizational repositories. Established life science data repositories, including PRIDE, the European Genome-phenome Archive (EGA) [46], MetaboLights [47], and Gene Expression Omnibus (GEO) [48], provide widely used examples of domain-specific infrastructure that enable standardized submission, long term storage, and reuse of proteomics, genomics, metabolomics, and transcriptomic data [49]. In addition to repositories, curated, and educational registries offer an informative infrastructure for decision making, interlinked descriptions of data policies from journal publishers and funding agencies [13]. According to most sponsors, data should be archived for at least ten years, but no recommendation has yet been made what to do with this data after the 10-year period [50].

In biotechnology data management, it is crucial to manage data-intense disciplines transparently and efficiently in order to safeguard valuable and sensitive data without redundancy in a secure and responsible manner [17, 51]. While In bioprocess technology, high-throughput data can be generated effectively using workflow pipelines or automated data handling [6, 52], in life science low and high throughput are feasible [17, 51]. Complex protocols in laboratory, proprietary data must be taken into account, especially in the case of low throughput in the laboratory, protocol deviations, and errors in the quality of the raw data [5]. Therefore, documentation of procedures, analyses, and data collection in all phases of the DLC are essential for responsible RDM [43].

A key aspect of biotechnology research involves heterogeneous diverse data sources from life science lab experiments, process engineering, or vendor specific equipment data formats. Alongside the contrast between high and low throughput, these data formats and amounts require significant effort to organize [5, 53]. Proprietary original data must be stored, archived, standardized and harmonized in process chains, as certain procedural steps and successive live scientific and engineering expressions often generate it [5]. Agile adaptation is essential for the harmonious processing of data such as OMICS, process or modeling data throughout the entire DLC as a workflow, it requires documentation in order to prevent misinterpretations [36, 37]. Heterogeneous data and data sets, such as those that inevitably arise in biotech research, require reproducible standards, such as the FAIR principles, standardized metadata schemas e.g., MIAME [54] or ISA-Tab, and controlled vocabularies and ontologies together with structured data management from the beginning of their life cycle. All models proposed to build on the FAIR consistent data life cycle: planning, generation, processing, storage, transfer, and reuse. However, this is challenging in the data-intense biotech sector, as data is generated in heterogeneous data formats through various analysis pipelines, different workflow engines, diverse devices, tools, and analytical software [55].

## From concept to practice

**Fig. 1:**
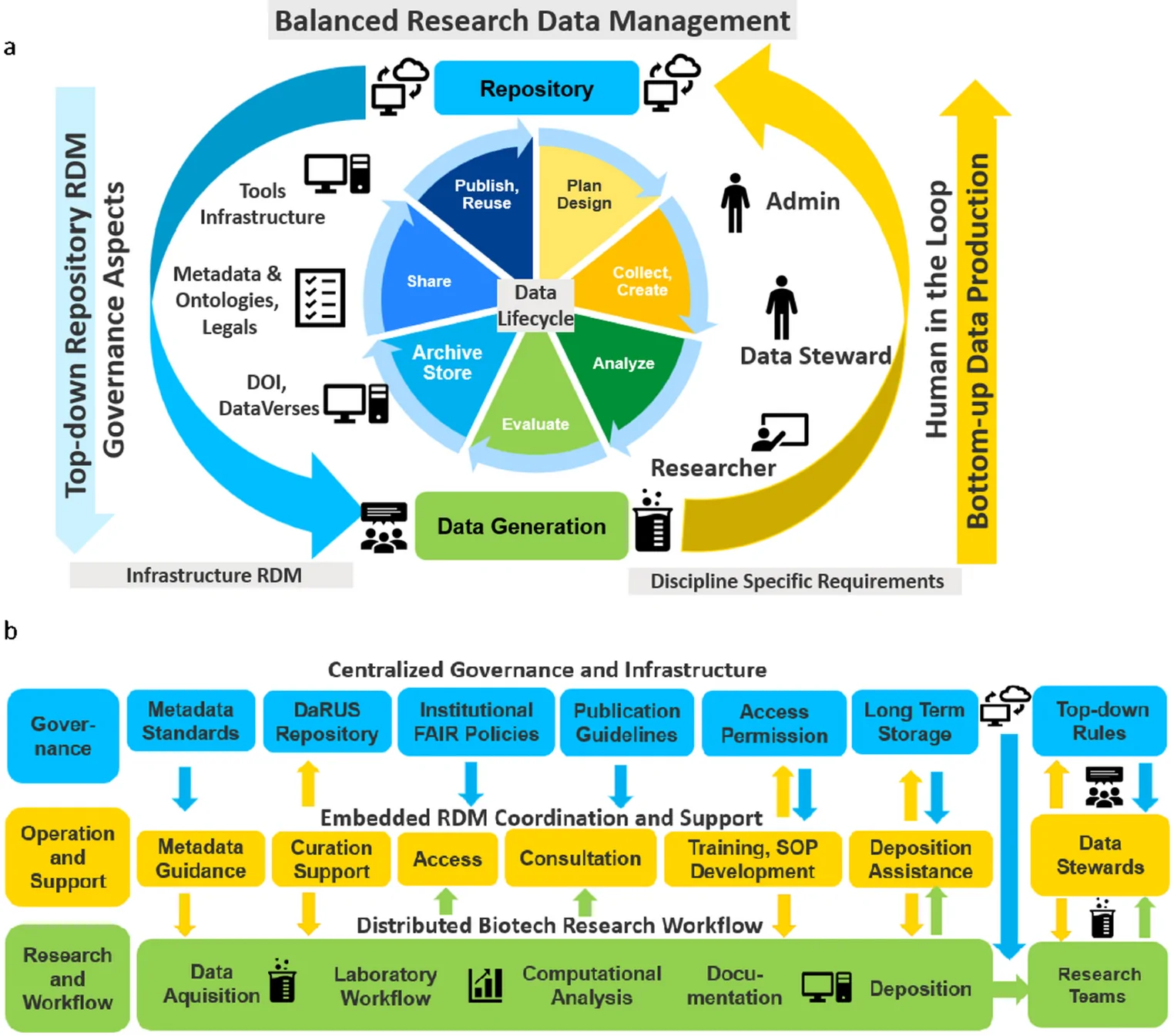
Illustration of an integrated top-down and bottom-up FAIR RDM based on the experiences of the SPP 2170 use case. The balanced model, which integrates both approaches, proved to be a practical FAIR-compliant data management solution in the use case by focusing on the data lifecycle. DLC adapted from [25]. Centralized governance infrastructure (blue), operational FAIR support (yellow), distributed biotechnology research workflows (green). **a** Governance and infrastructure was provided by the institutional repository (top-down) with templates of metadata, licensing, identifiers. Data generation (green) is centered around the data life cycle, and discipline specific requirements (bottom-up). **b** Interaction between governance (blue), operational support (yellow) and distributed biotechnology research workflow (green). The institutional repository provides standardized metadata, licensing, and identifier frameworks, while heterogeneous experimental, modeling, and simulation workflows require additional support. Data stewards (yellow) mediate between researchers and repository administrators to implement necessary adjustments such as metadata extensions, data format conversions, and access control configurations

In a use case involving 50 international researchers over a six-year period, we gained practical experience in adressing requirements such as long-term archiving, metadata documentation, the managment of heterogeneous and proprietary data, and documentation throughout the data lifecycle. Throughout this process, ensuring both user-friendliness and compliance with governance standards were equally important for effective biotechnology data management (Fig. 1). A central institutional infrastructure repository (DaRUS) was used (Fig. 1a) and complemented with operational discipline-specific FAIR support within the research workflows for biotechnology researchers (Fig. 1b) from two different groups, national and international researchers across Germany. The researchers were not recruited specificially for this study. Rather the presented use case is based on the DFG-funded priority program SPP2170, which consisted of 10 collaborative projects (“tandem teams”) from multiple institutions in bioprocess engineering across Germany. In addition, RDM-related experiences from institutional early stage researchers informed the practical implementation perspective. Our contribution is not intended as a representative survey of the broader biotechnology community, but as an empirical grounded perspective derived from accompanying researchers over multiple years and across different teams.

### Use case context and implementation settings

#### Implementation

The institutional repository (DaRUS), a Harvard implementation was provided by the University Research Data Infrastructure. Translated, operational FAIR implementation was mediated through distributed RDM support and tailored to research teams and computational workflows. Within the use case, domain-specific FAIR support were integrated into biotechnology research workflows. This included access for researchers at eight additional participating universities, metadata updates, ORCID integration, and the implementation of discipline-specific schemas such as EngMeta for engineering-related datasets. The repository was institutionally governed and has been used by the SPP Network since 6 years and the institutional team since 3 years.

#### Collection methods and metrics

This study draws on longitudinal operational observations from six years of RDM support and provides a practice-based perspective on the organizational and technical implementation of FAIR-oriented RDM rather than a quantitative evaluation. FAIR alignment was assessed through indicators such as metadata completeness, repository accessibility, persistent identifier, access management, documentation practices, and interoperability support.

#### Researchers expectations and training

Researchers expectations were captured through iterative qualitative engagement during onboarding meetings, project coordination discussion, support requests, and requrring training interactions rather than formal questionaires. A growing need for consultation, access concepts for publications, and the provision of data under specific licensing conditions to protect ongoing patent applications highlighted the demand for user-friendly support. This was addressed through self-learning courses and SOPs, and targeted training formats (onsite and online workshops) complemented by on-demand consultatins with data stewards.

Training, SOPs, and guidance have been provided since the start of the project, and additional data repositories have been introduced to meet users’ needs, even when dealing with sensitive data. These activities included also metadata guidance, curation support, repository data deposition assistance, and consulting regarding access and sharing decisions across multiple stage of DLC. The FAIR provision of data and repository deposition in the last data lifecycle demonstrated that a shift in thinking was necessary here. The documentation and storage of data—not just in proprietary data formats—must be taken into account from the very beginning of the data lifecycle.

### FAIR-by-design: complementing governance with user requirements

Whereas governance mechanisms provide centralized standards, repository infrastructure and institutional policies (metadata standards, access control mechanisms, compliance structures) there is an increasing need of the researchers on adapting FAIR implementation to heterogeneous and evolving research workflows and integrate it into the day-to day-live. In the use case, limitations arose when only the infrastructure was provided:

- Lack of acceptance of overly generic metadata schemas by researchers,
- Incompatibility with proprietary file formats generated by devices,
- Varying requirements regarding the data sensitivity of the projects.
- Insufficient flexibility when big data from subfields/subprojects needs to be stored,
- As well as resistance from researchers toward data workflows perceived as an administrative burden.

### Complementing top-down with bottom-up requirements

In this use case the top-down approaches include shared interoperability frameworks, metadata governance, and persistent identifier structure, whereas bottom-up approaches involve the community-driven evolution of research worksflow, metadata extensions, and domain-specific semantics (Fig. 1). The bottom-up FAIR implementation (operational adaption, curation support, workflow translation, metadata mediation), researcher guidance) additionally refers to researcher-centered operational support activities embedded within day-to-day laboratory practice. These activities included workflow-specific metadata guidance, iterative data curation support, data naming conventions, assistance with repository deposition, and consulting regarding data access and sharing decisions across multiple stages of the DLC.

The use case combined top-down governance with bottom-up flexibility. Top-down governance ensured compliance with regulatory frameworks, consideration of intellectual property requirements, and the FAIR principles through standardized metadata and shared infrastructure. This established a reliable foundation for reproducibility, data integrity, cross-project interoperability, and legal compliance. Complementing this, bottom-up approaches allowed researchers to adapt workflows and metadata practice to projects-specific needs through metadata adjustments, customized documentation fields, storage options for proprietary data, and case-by-case access control (Fig. 1a).

#### Data stewards

However, user needs were often overlooked in top-down approaches. Through bottom-up involvement, research groups were able to adapt workflows, data structures, and formats to specific project requirements. Data stewards are the “middle layer” to translate governance, rule, metadata standards, institutional FAIR policies, long term storage data guidelines to user and researcher. The bottom-up formulated requirements of the users were also supported via metadata guidance, curation support, access control, SOP guidance, and depositions assistance for data (Fig. 1b). The combination of top-down and bottom-up approaches with additional training additionally reduces uncertainty for researchers and accelerates the adoption of FAIR practices (Fig. 1). In order to optimize data management, the entire DLC had to be considered: from planning and collection to documentation and publication. Interdisciplinary data formats from the life sciences and engineering fields were taken into account, by avoiding proprietary formats. This required additional resources, with a dedicated coordinator managing data access, metadata templates, workflow communication, and standard operation procedures (SOP).

### Outcome and added value

Metadata Templates, provided through governance processes combined generic fields (e.g., tittle, authors, description, keywords, funding information, persistent identifier) with discipline-specific extensions adapted to different biotechnology workflows. In the use case, these were expanded using EngMeta templates. Adjustment and refinements of metadata practice were discussed iteratively with repository administrators, data stewards, and researchers during the support process. Rather than developing new metadata standards, our contribution focused on adapting existing repository-supported metadata structures and improving their practical usability for researchers (Fig. 1b).

#### Access and collaborative data handling

To facilitate collaborative data handling, participating institutes were integrated into the DaRUS repository, which provided the general infrastructure. For the use case, eight additional universities were integrated, that previously did not have access. Through cooperation with the partners’ IT departments, protocols were integrated, enabling users to log in via university accounts.

#### Controlled interoperable access

Persistent identifiers, such as Digital Object Identifiers (DOI) and Handles provide stable referencing and lightweight interoperability linking to detailed domain-specific documentation. In the use case, these contributed to enabling cross-project integration, feedback, and openness while simultaneously protecting sensitive data through controlled access (e.g., CHO-patented mammalian cells-Dataverse).

#### Heterogenous and proprietary data

The main implementation challenge resulted from the heterogeneity of biotechnology data and workflows across modelling (e.g., Matlab data), laboratory experimentation (excel, python, devise specific data) fermentation process (process data), and genetic engineering (omics, sequences). Research groups differed substantially in data structure, file organization, metadata requirements, and documentation practice. These issues were adressed through iterative support by the data stewards (Fig. 1b) including hands-on consultation, workflow guidance trainings, SOP development. Direct assistence was provided in cases where SOPs were not practicabel, or researchers hesitated. The teams of the use case used a variety of proprietary and instrument- specific file formats generated by laboratory devices, modelling software, imaging systems, sequencing workflows, and process engineering platforms. Because these formats varied substantially between projects, no single standardized conversion workflow was feasible. Whenever possible, proprietary formats were complemented by open or interoperable export formats (csv). Where direct conversion was not practical, the original files were preserved alongside descriptive metadata and contextual documention, that often needed direct consultation and effort by the data steward support team (Fig. 1b).

#### Workflow communication

Because many project-specific questions emerged during implementation workflows supplementary SOP documents were developed and communicated addressing topics such as handling of heterogeneous data formats, large scale data uploads, and metadata documentation. Researchers received training and hands-on support during dataset publication and repository review processes.

#### Onboarding and training

Structures and recurring training (workshops, online sessions, and practical exercises= covered repository use, metadata, FAIR principles, and publication workflows. These were complemented by self-learning resources, including Dataverse training environments for early-stage researchers. Continued support requests highlighted the need for discipline-specific, hands-on training to enable effective RDM implementation.

## Translating FAIR principles into biotechnology RDM practice

Our six years of experience implementing FAIR-oriented RDM in biotechnology demonstrate the FAIR principles are as relevant in biotechnology as in other scientific disciplines, but their practical application requires more than technical infrastructure alone. It suggests that the primary limitation of FAIR implementation in heterogeneous biotechnology environments is not the absence of repository infrastructure, but the difficulty to translate generic FAIR requirements into workflow-specific research practice. Effective FAIR research data management extends beyond technical, governance tasks and encompasses both stakeholders as human in the loop and technical AI-driven machine-readable data [6]. Research domains commonly rely on heterogenous ecosystems of historically evolved repositories shaped by institutional and disciplinary requirements. Accordingly, the repository discussed here is intended as a use case srather than a universal infrastructure model, highlighting broader challenges in interoperability, governance, and metadata stewardship across distributed FAIR ecosystems. Many of the complexity challenges described here, including evolving data formats, changing workflows, and semantic shifts in ontologies, are not unique in biotechnology but are common across dynamically developing domains. While increased openness and reusability of biotechnology data offer significant benefits it also place higher demands on governance [36]:

i. Determining how long research data should remain openly accessible remains an unresolved challenge in FAIR-compliant RDM, requiring a balance between, long-term scientific value [50], and practical constraints such as privacy protection, data protection, regulations and infrastructure cost.
ii. Although international legislation [56] and EU regulations [18] exist, these are often difficult to understand due to their abstract language. Concrete instructions or action plans are often lacking. Existing legal and institutional frameworks often lag behind rapid data disclosure in science, leading to ambiguities in access control and responsible use, referred as regulatory gaps [37]. Strict requirements already exist in sectors, such as healthcare, including handling of patient and health data, such as European Health Data Space (EHDS) which address the processing of health data, data minimization, consent, and security measures for interoperable data exchange, all aimed at protecting sensitive personal data and individual privacy [57]. This involves algorithm-to-data approaches, in which analytical algorithms are applied to the data rather than transferring data to external systems. This enables secure analysis of sensitive datasets on-site while minimizing data protection risks and data movements [58].
iii. Data sets may enable unintended downstream applications or pose risks outside their original research context. Various risk categories, including open access to detailed experimental protocols may be misused for unauthorized reuse, misinterpretations or harmful purposes, including biosecurity [36, 37].
iv Without robust standards, comprehensive metadata and effective curation, expanded data sharing can increase the risk of misuse or misinterpretation. Research data management must go beyond technical, top-down tasks and machine-readable formats and take into account the use of data by stakeholders, citizen scientist, AI and other users in order to maintain data integrity and quality [46, 59].
v Reuse of human-derived datasets (e.g., genomic data) may conflict with ethical concerns, creating governance challenges related to data protection, privacy and consent [34].
vi Documentation is often the most critical element of FAIR data, as it provides the contextual information required to interpret and reuse datasets correctly. Without complete and standardized documentation, even structured data remain difficult to understand, reproduce, or validate. However, documentation is frequently incomplete across DLC or linked to proprietary systems, limiting accessibility and interoperability. In FAIR publication, explicit documentation of data provenance, processing steps, and references enable transparent assessment of how the data supports scientifically valid conclusions and facilitates producibility and reuse [37].
vii With the increasing influence of online platforms and social media, biological and personal data may be repurposed beyond their original scientific intent, for example through re-identification, secondary commercial use, profiling, or unauthorized data linkage across platforms. Misinterpretation of biomedical (personal) data, ecological data, and geoprivacy are already reported [36, 60].

In practice, sets of guidelines for handling research data can be established through a robust technical infrastructure and clear top-down management. This makes it possible to comply with security standards, guidelines for handling sensitive data, metadata, and security regulations for research data, while also raising awareness of potential incidents [61].

But risk mitigation by each individual researcher is also crucial to ensure that FAIR data practices in biotechnology are applied in a trustworthy and responsible manner [37]. Bioprocess data can be rendered FAIR without compromising regulatory compliance or trade secrets. This requires comprehensive metadata that describes experiments without revealing critical process know-how, as well as the use of standardized ontologies, the deployment of repositories with controlled access, and the implementation of role-based access controls [62].

The main implementation challenges in the use case resulted from the heterogeneity of data and workflows across in biotechnology across modelling, laboratory experimentation, fermentation processing and genetic engineering. The research teams of the use case differed substantially in data structure, file organization, metadata requirements, and documentation practice. As a result, generic top-down RDM guidelines were perceived as too abstract for daily research workflows. Additional challenges included incomplete metadata, inconsistent file naming, or limited researcher familiarity with FAIR principles, and time constraints, particularly under publication pressure.

Researchers should be aware that publishing data in repositories only in the final phase of the life cycle can complicate storage due to insufficient documentation and must therefore be taken into account in workflows as well as in the planning of experiments and studies right from the start of the life cycle [43]. With the support of data stewards’ adjustments were made to experimental workflows, and user-friendly storage of biotechnology data formats was achieved while adhering to governance standards familiar from other disciplines [36]. Support, communication, workflow adjustments, and RDM training helped engage researchers and ensure data quality throughout the entire data life cycle [25].

### Governance approach in the use case

While a top-down strategy requires clearly defined overarching goals from the outset, it offers advantages such as consistency, governance, an integrated perspective, standardized directory structures [63] and secure deposition of data. However, it can potentially be inflexible and costly in terms of infrastructure for long-term archiving and personnel expenses [64]. Furthermore, the standardization and harmonization of data is resource- and time-intensive [63, 65] as manual metadata curation, alignment with controlled vocabularies and ontologies, data cleansing, and iterative quality control and validation are required to ensure consistency across heterogeneous datasets [54]. The FAIR principles are as relevant to biotechnology as they are to other disciplines, making responsible data management essential. The use case has shown, that repositories provide governance, licenses, metadata guidelines, and templates for data documentation [66], but researchers are reluctant to adopt them. Effective RDM requires practical guidelines that balance openness with necessary restrictions on data access. Without such guidance, researchers may struggle to identify sensitive data early in the data life cycle, comply with ethical, legal and security requirements, and determine appropriate publication strategies. In this context, data stewards play a crucial role by supporting institutional governance, while providing user-oriented guidance that helps researchers manage the complexity in RDM in interdisciplinary biotechnology environments and ensures the long-term preservation and reuse of research data.

### User-friendly perspectives in the use case

Complementing bottom-up strategy offers flexibility for an efficient adoption of special use cases and makes it easier to integrate pipelines for project data [64]. The disadvantage, however, is a higher integration effort and the inability to prevent silos, which often arise from specific applications [67] and a case by case decision for regulatory compliance. The design of RDM from bottom-up can adapt infrastructure or institutional strategy in an agile way, while using the organizational capacity through iterative combination [65]. Subsequently, this required intensive coordination and tool support from data steward and technical staff [34, 65]. Additionally, it shifts the responsibilities to the researchers, they in return need more training and capacities. Use cases that are adapted by researchers, and administrators throughout the entire data life cycle therefore provide and need an important basis of experience [19]. In our use case we found that the benefits of the top-down approach (framework conditions, metadata guidelines, and templates for data documentation) are best combined with a user-friendly bottom-up approach in order to leverage the advantages of both. However, this requires the creation of an active support structure that provides consulting, training, and motivation.

## Outlook

Future efforts should focus on integrating subject-specific, often heterogeneous requirements more closely into institutional infrastructures from the outset. Adopting this approach will enable the expected dynamics and changes in data management to respond to dynamic changes in research and society. Furthermore, this process should be iteratively integrated into the agile workflow throughout the entire data life cycle. Responsible improvements can then be realized in relation to modern requirements for automation, data quantities and interoperability in biotechnology. In view of the growing complexity of research and the increasing volume of biotechnology data, it is crucial to promote high-quality data across projects. This can be achieved by supporting a robust technical infrastructure and by strengthening the biotechnology researchers through ongoing research data management training.

This perspective highlights that the practical implementation of FAIR data management in dynamic research environments extends beyond repository availability and standardized metadata frameworks. Our longitudinal operational experience showed that heterogeneous laboratory workflows, metadata complexity, and governance ambiguity, frequently limited the effectiveness of purely technical approaches. Sustainable FAIR adoption therefore depended on continuous, discipline-specific and human-in-the-loop support embedded directly within research practice.

These observations complement existing framework-oriented FAIR literature by emphasizing the operational and organizational conditions required for long-term implementation in real-world biotechnology environments. Our findings highlight the important role of data stewards. By supporting compliance while maintaining user-friendly workflows, data stewards help researchers navigate the complexity of interdisciplinary biotechnology research and contribute to the long-term preservation, accessibility, and reuse of research data.

Practical application Our practical experience demonstrates that successful implementation of FAIR-oriented RDM in biotechnology requires balancing data opened with ethical, legal, and security constraints. The findings highlight the role of data stewards as intermediaries between institutional governance and research practice, supporting both compliance and user-friendly workflows. This function is particularly important in interdisciplinary biotechnology environments, where heterogenous data types, standards, and regulatory requirements increase the complexity of sustainable research data management.

## Data Availability

No datasets were generated or analysed during the current study.
